# Different responsiveness to a high-fat/cholesterol diet in two inbred mice and underlying genetic factors: a whole genome microarray analysis

**DOI:** 10.1186/1743-7075-6-43

**Published:** 2009-10-17

**Authors:** Mingzhe Zhu, Guozhen Ji, Gang Jin, Zuobiao Yuan

**Affiliations:** 1 Institute of Health Sciences, Shanghai Institutes for Biological Sciences, Chinese Academy of Sciences & Shanghai Jiao Tong University School of Medicine, Shanghai, PR China; 2 Shanghai Institute of Organic Chemistry, Chinese Academy of Sciences, Shanghai, PR China; 3 ShanghaiBio Corporation, 675 US Highway One, North Brunswick, NJ, USA; 4 Shanghai Biochip Co., Ltd and National Engineering Center for Biochip at Shanghai, Shanghai, PR China; 5 Department of Surgery, University of Iowa, Iowa City, USA; 6 Graduate School of the Chinese Academy of Sciences, Shanghai, China

## Abstract

**Background:**

To investigate different responses to a high-fat/cholesterol diet and uncover their underlying genetic factors between C57BL/6J (B6) and DBA/2J (D2) inbred mice.

**Methods:**

B6 and D2 mice were fed a high-fat/cholesterol diet for a series of time-points. Serum and bile lipid profiles, bile acid yields, hepatic apoptosis, gallstones and atherosclerosis formation were measured. Furthermore, a whole genome microarray was performed to screen hepatic genes expression profile. Quantitative real-time PCR, western blot and TUNEL assay were conducted to validate microarray data.

**Results:**

After fed the high-fat/cholesterol diet, serum and bile total cholesterol, serum cholesterol esters, HDL cholesterol and Non-HDL cholesterol levels were altered in B6 but not significantly changed in D2; meanwhile, biliary bile acid was decreased in B6 but increased in D2. At the same time, hepatic apoptosis, gallstones and atherosclerotic lesions occurred in B6 but not in D2. The hepatic microarray analysis revealed distinctly different genes expression patterns between B6 and D2 mice. Their functional pathway groups included lipid metabolism, oxidative stress, immune/inflammation response and apoptosis. Quantitative real time PCR, TUNEL assay and western-blot results were consistent with microarray analysis.

**Conclusion:**

Different genes expression patterns between B6 and D2 mice might provide a genetic basis for their distinctive responses to a high-fat/cholesterol diet, and give us an opportunity to identify novel pharmaceutical targets in related diseases in the future.

## Background

Western-type/high-fat diets are a well-established risk factor for both atherosclerosis and gallstones, due to the consequent high levels of circulating [1] or biliary cholesterol [2]. The liver is an essential organ in maintaining cholesterol homeostasis because of its capability to clear excessive cholesterol from blood through direct excretion into the bile [3] or the bile acid synthesis pathway [4]. Alteration in cholesterol homeostasis may lead to accumulation of excessive cholesterol in bodies and deleterious consequences. Elevated blood cholesterol levels, especially LDL, will eventually lead to atherosclerosis [5]. At the same time, hypersecretion of cholesterol into bile is a prerequisite for gallstones formation [6]. In addition, accumulating data have suggested that genetics plays a pivotal role in cholesterol homeostasis and subsequent gallstones formation and atherosclerosis. For instance, mutation of ABCG5/G8 (ATP-binding cassette, sub-family G (WHITE), member 5/8) limits biliary excretion of sterols and alters susceptibility to gallstones and atherosclerosis [7].

Inbred mice strains have been a powerful force in elucidating the genetic basis of human physiology and pathophysiology [8]. When fed to a high-fat/cholesterol diet, inbred mice strains show varying susceptibilities to atherosclerosis and gallstones respectively. C57BL/6J (B6) mice are susceptible and DBA/2J (D2) mice are resistant to both atherosclerosis and gallstones [9-11]. The analysis of differences in the responsiveness among inbred strains to high-fat/cholesterol diets is leading to new insights into associated diseases including atherosclerosis, diabetes, obesity and gallstones [11]. Although researchers have exerted great efforts using genetic tools to illuminate the genetic factors underlying the different responses to high-fat/cholesterol diets between inbred mice strains [12], the mechanisms have not been clearly elucidated. In the past decades, microarray technology has provided a powerful tool to study genetic and molecular events in complex diseases [13]. Here, studies were designed to examine the dynamically different responses to a high-fat/cholesterol diet between C57BL/6J (B6) and DBA/2J (D2) inbred mice, and uncover the underlying genetic factors using Affymetrix microarray technology as well. This may provide us an opportunity to predict the risks of developing high-fat diets related diseases, and construct a basis for identification of candidate genes in humans.

## Methods

### Animals and Diets

Six weeks-old B6 and D2 female mice were purchased from Shanghai SLAC Laboratory Animal CO. LTD, China, and maintained in temperature and humidity-controlled room. Mice were fed a high-fat/cholesterol diet (0.5%cholic acid, 1.25%cholesterol and 15%fat) for 0(control), 1, 4, 12 and 21 weeks respectively, with 10 mice of each strain for each time point. Blood was collected through inferior cava veins. Biliary bile was aspirated from the gallbladder with sterile injectors. All procedures were approved by the Institutional Animal Care and Use Committee of Shanghai Institutes for Biological Science.

### Serum and bile cholesterol assay

Serum and bile total cholesterol (TC), HDL-cholesterol and Biliary total bile acid levels were assayed using a Total Cholesterol Kit, HDL-Cholesterol Kit and Total Bile Acids Kit (Shenzhen Mindray Bio-Medical Electronics Co., Ltd, China). Serum cholesterol esters were assayed using High Performance Liquid Chromatography (HPLC) in Shanghai Institute of Organic Chemistry, Chinese Academy of Sciences.

### Evaluation of atherosclerotic lesions and gallstones

Hearts containing upper sections of the aorta were collected and evaluation of aortic lesions was performed using the Oil-red O method described by Beverly Paigen [14]. Briefly, every other 10 μm section of aortic sinus was fixed on polylysine-coated microscope slides and stained with Oil-red O. Aortic surface lesion sizes were evaluated by using the grid on the microscope eyepiece and presented as μm^2^. Gallstones formation was detected by naked eyes and described as the percentage of stone presence among mice of the same group. Gallstones in the same group at each time-point were pooled and weighed.

### Liver tissues RNA Extraction and microarray experiments

Liver tissues were removed from mice and stored in liquid nitrogen until use. Total RNA was extracted by using Trizol reagent (Invitrogen) according to the manufacturer's instructions, and purified by using a RNeasy Mini Kit (Qiagen). RNA samples of each group were then used to generate biotinylated cRNA targets for the Affymetrix GeneChip Mouse Genome 430 2.0 Array, which contained 39000 transcripts. All experiments were performed by following the protocol of Affymetrix Inc.

After hybridization, arrays were stained in the Fluidics Station 450 and scanned on the Affymetrix Scanner 3000. Fluorescent signal intensities for all spots on the arrays were analyzed using the Gene Chip Operating System (GCOS; Affymetrix). Ratios were calculated between 0 week and the rest of time points within the same mouse strain, and then between two strains at each of five time points. Genes with a fold change of at least 2 were selected for further analysis. Hierarchical analysis was performed using Cluster 3.0 to define the genes expression patterns. Furthermore, the selected genes were grouped in functional categories based on Gene Ontology database (GO: ), and functional pathways (KEGG and BIOCARTA) were also analyzed by using DAVID 2007.

### Quantitative real-time polymerase chain reaction (QRT-PCR)

To validate microarray data, QRT-PCR was performed for every mouse at each time point of two strains. It was operated on an ABI 7900 instrument with gene-specific primers and SYBR Green PCR Master Mix (Applied Biosystems) according to the manufacturer's recommendation. Reaction conditions are available on request. The PCR primers used in this study are listed at Table S1 in Additional file 1.

### TUNEL Assay and Western blot analysis

Apoptosis of liver cells was detected by immunohistochemical TUNEL (Terminal Transferase dUTP Nick End Labeling) assay using in situ cell death detection kit, Fluorescein (Roche). Formalin-fixed liver tissues were embedded in OCT compound for cryostat sectioning, and 5-μm sections were fixed on polylysine-coated microscope slides for TUNEL assay according to the manufacturer's instructions. Sections were analyzed under a fluorescence microscope (objective ×20). Positively stained nuclei of liver cells were counted for analysis.

Liver tissues were homogenized and proteins were extracted by using RIPA Lysis Buffer (Beyotime Institute of Biotechnology, China) containing Phosphatase Inhibitor Cocktail Tablets (Roche) according to the manufacturer's instructions. Western blot was performed according to the methods described previously [15]. Antibodies to Hspa1b, Parp and Cyp7a1 were commercially obtained from Abcam (Cambridge, MA), Cell Signaling Technology (Beverly, MA) and Santa Cruz Biotechnology, separately.

### Statistical Analysis

All values were presented as mean ± SD. Statistical analysis was performed using ANOVA and the Student's t test, and p < 0.05 was considered significantly different.

## Results

### Dynamic distribution of cholesterol in serum and bile and bile acid yields

**Figure 1:**
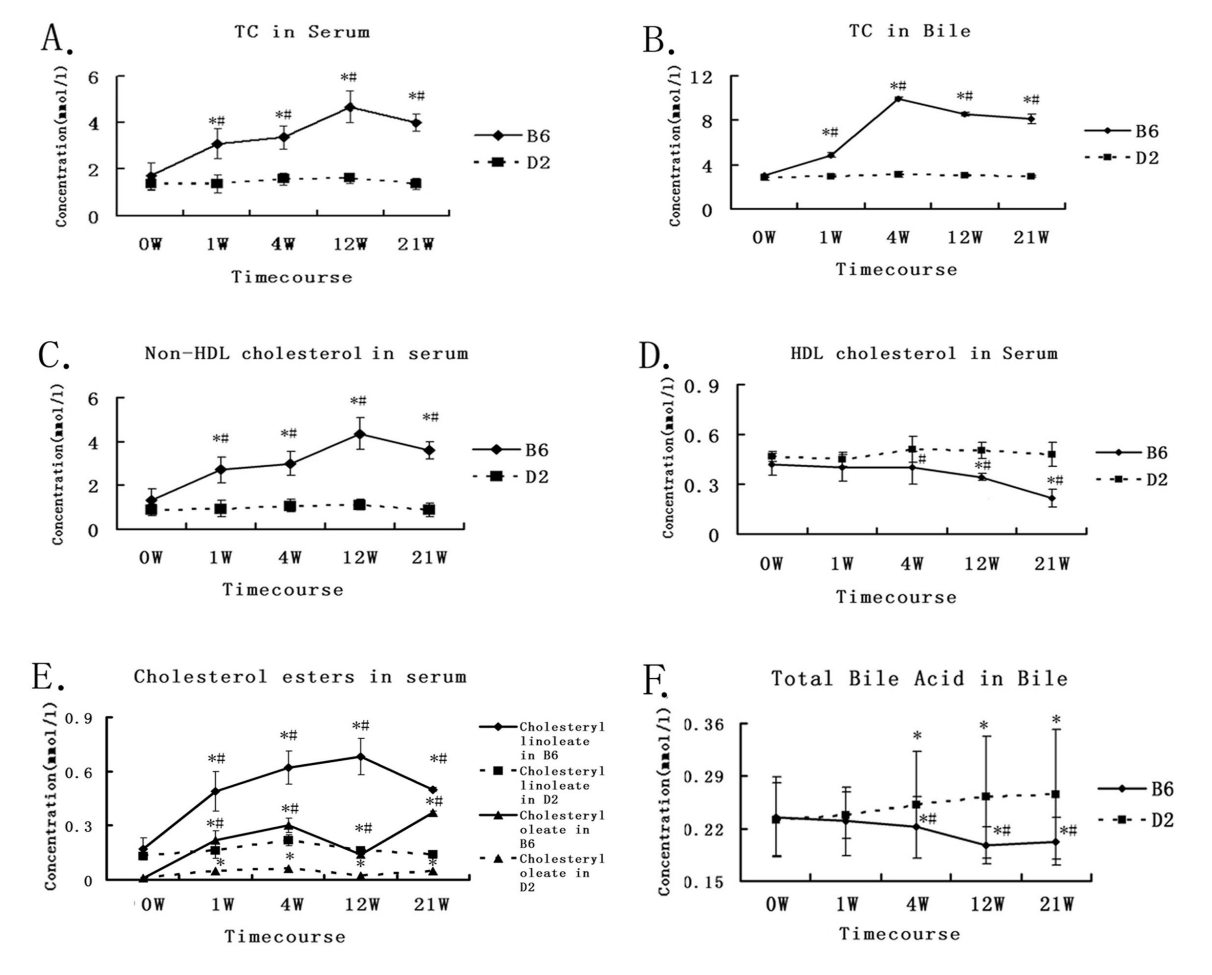
**Dynamic distribution of cholesterol and bile acids**. The x-axis represents each time point. The y-axis depicts concentrations (mmol/l). Solid lines represent data in C57BL/6J (B6) mice and dashed lines represent data in DBA/2J (D2) mice. *P < 0.05 (compared to 0^th ^week), #P < 0.05 (compared to D2 mice); ***A*: **Total cholesterol (TC) in serum; ***B*: **Total cholesterol (TC) in bile; ***C*: **Non-HDL cholesterol in serum; ***D*: **HDL cholesterol in serum; ***E*: **Cholesterol esters (Cholsteryl oleate and linoleate) in serum; ***F*: **Total bile acids in bile.

As shown in Figure 1, after fed the high-fat/cholesterol diet, serum total cholesterol (TC) levels in B6 mice increased as early as the 1^st ^week and reached their peaks at 12 weeks. Non-HDL and cholesteryl linoleate cholesterol levels also reached their peaks at 12 weeks. However, the HDL cholesterol level decreased after the 12^th ^week. In contrast, these cholesterol levels did not significantly change in D2 mice.

As total cholesterol levels in serum increased, the bile total cholesterol levels in B6 mice also significantly increased as early as the 1^st ^week, with its peak at four weeks. However, biliary bile acid levels decreased in B6 mice and increased in D2 mice after the 4^th ^week.

### Gallstones and atherosclerotic lesions development

The two strains considerably differed in the prevalence of gallstones. We observed that 10% of B6 mice developed gallstones as early as the 4^th ^week, when bile cholesterol concentration had reached its peak, while bile acids decreased after. The gallstone percentage increased to 80% at 12 weeks and reached 100% at 21 weeks, when bile cholesterol was maintained at higher levels. The weights of gallstones were also distinctly increased after the 4^th ^week. However, atherosclerotic lesions were observed only at 21 weeks. Of note, Oil-red O stained lesions (7800 ± 211 μm^2^) were present in aortas of all B6 mice at 21 weeks. In contrast, neither atherosclerotic lesions nor gallstones formation was detected in D2 mice at any time point. See Figure S1 in Additional file 2 for details.

### Gene expression profile and pathway analysis

**Figure 2:**
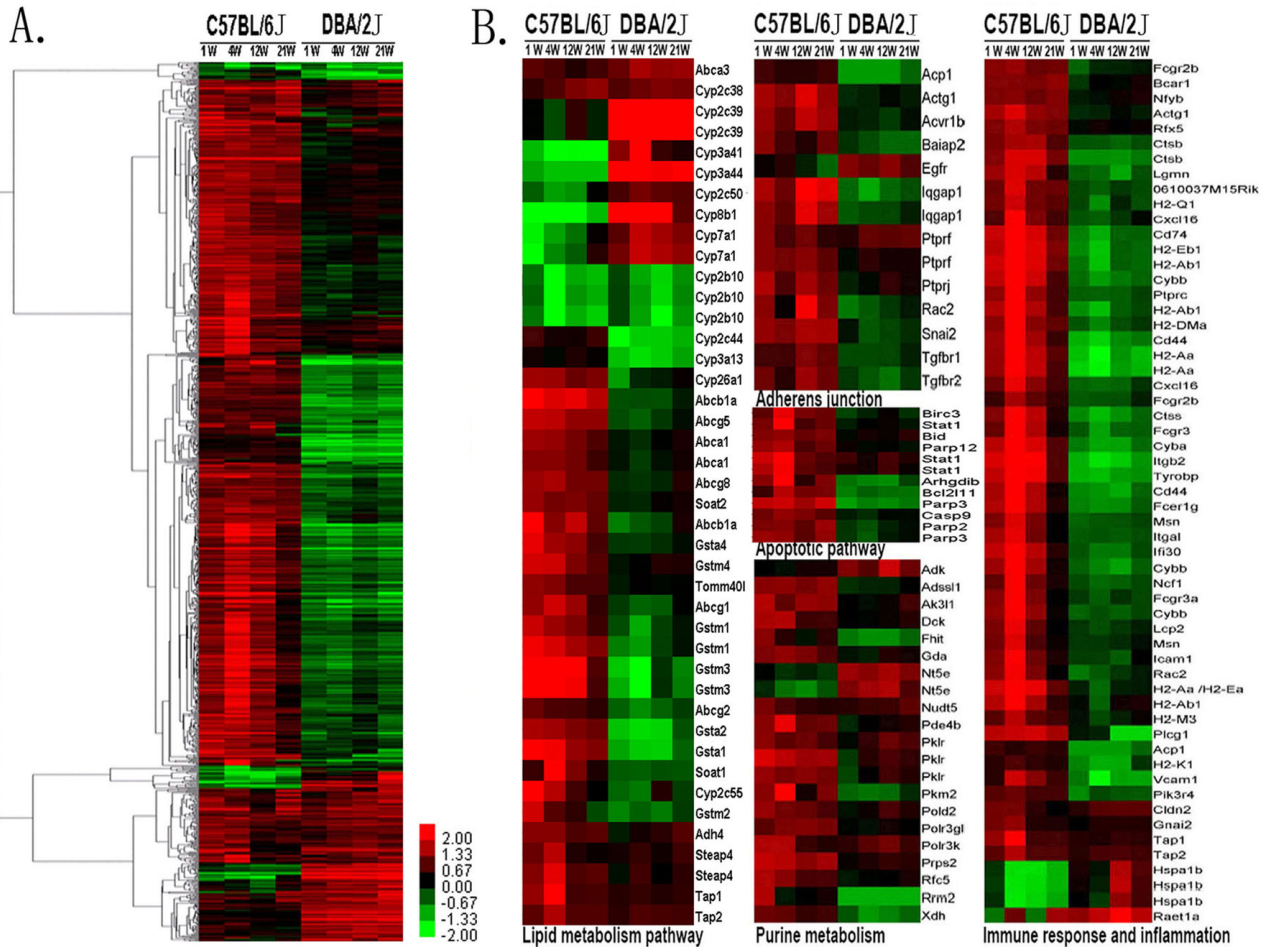
**Hierarchical cluster of differentially expressed genes between B6 and D2 mice**. All the relevant genes are grouped by hierarchical clustering based on expression values (log_2_ratios) across all the samples. Ratios were calculated between 0 week and the rest of time points in each mouse strains. Samples (Four time-points in C57BL/6J and DBA/2J, respectively) are displayed in columns and genes in rows. Gene expression is represented as a color, normalized across each row, with brighter red for higher values and brighter green for lower values. ***A***. Heatmap of all the differentially expressed genes, most of which have different expression patterns between B6 and D2; ***B***. Heatmap of genes involved in some major pathways, gene symbols listed to the right.

Using the 2-fold change cutoff, we identified 1191 genes for further analysis including 1051 known genes and 140 ESTs. Detailed information of these genes was listed at Table S2 in Additional file 3. The overall functional categories of these genes were grouped into transcription regulation, protein modification, transport, cell cycle/adhesion/apoptosis, signal transduction, immune response/inflammation, lipid metabolism, cell metabolism, development, RNA processing and others or unknown function (See Figure S2 in Additional file 4). Using hierarchical cluster analysis, we observed that the two inbred mice strains had distinctly different genes expression patterns. Most of the genes up-regulated or down-regulated in B6 mice might have the opposite patterns in D2 mice (Figure 2A).

To examine whether there was recognizable biological relevance to the genes expression patterns, further functional pathway analysis was performed. Using the combination of GO, KEGG and BIOCARTA, we observed that genes were involved in several key functional pathway groups such as lipid metabolism, oxidative stress, immune response/inflammation and cell apoptosis.

We observed that multiple lipid metabolism genes were differentially expressed between B6 and D2 mice. For instance, genes *Abca1 *(ATP-binding cassette sub-family A member 1)*, Abcg5, Abcg8 *and *Soat2 *(sterol O-acyltransferrase2) were up-regulated in B6 mice but unchanged in D2 mice. *Soat1 *(sterol O-acyltransferase1) were up-regulated in B6 mice but down-regulated in D2 mice.*Cyp7a1 *(cytochrome P450 family 7 subfamily a polypeptide 1) and *Cyp8b1 *(cytochrome P450 family 8 subfamily b polypeptide 1) were down-regulated in B6 mice but up-regulated in D2 mice.

We also found that batches of oxidative stress relevant genes represented different expression patterns between B6 and D2. *Nox4 *(Nadph oxidase4)*, Homx1 *(heme oxygenase 1) and *Saa*(serum amyloid A) were up-regulated in B6 mice but down-regulated in D2 mice, whereas,*Hspa1b *(heat shock protein 1b) was down-regulated in B6 mice and up-regulated in D2 mice. Moreover, a number of genes involved in immune response and inflammation signaling pathways such as T cell receptor signaling pathway, leukocyte transendothelial migration, MHCI and MHCII pathways were altered, and most of these genes were up-regulated in B6 mice and down-regulated in D2 mice. Meanwhile, abundant apoptosis pathway genes such as *Bcl2-like 11*, *Bid *(BH3 interacting domain death agonist), *Casp9 *(caspase9) and *Parp *were also differentially regulated.*Bcl2-like 11 *and *Parp *were up-regulated in B6 mice and down-regulated in D2 mice. *Bid *and *Casp9 *were up-regulated in B6 mice but not altered in D2 mice.

Except for the above mentioned major pathways, genes expression in adherens junction and purine metabolism pathways was also differentially altered between B6 and D2. The heatmap of modulated genes involved in functional pathways was shown in Figure 2B. More information about detailed pathways and their corresponding genes were listed at Table S3 in Additional file 5.

Finally, to exclude the underlying differences prior to feeding and the influence of age factor on the gene expression levels, we also compared genes expression between B6 and D2 mice at the same time-point. We observed dominant expression patterns at all four time-points after 0 week. Most genes were up-regulated and few were down-regulated. The result coincided with our above findings (See Figure S3 in Additional file 6).

### Confirmation by quantitative real time PCR

**Figure 3:**
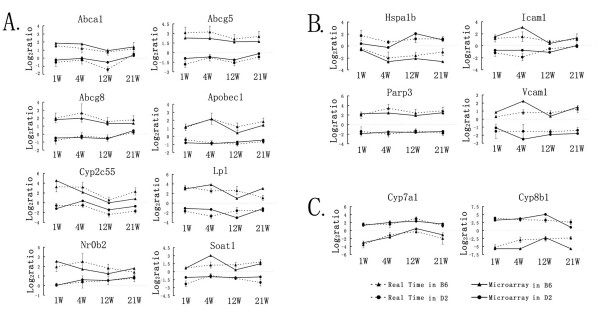
**Real time PCR validation of regulated genes in microarray**. Data were represented in three groups: ***A*: **cholesterol metabolism genes; ***B*: **inflammation and apoptosis genes; ***C*: **bile acid synthesis genes. The horizontal axis represents time points and the vertical axis represents log_2_ratio of each time point against 0 week in microarray and Real-time PCR data. Solid lines represent microarray data and dashed lines represent Real-time PCR data; marked with black triangles were data in C57BL/6J (B6) mice and marked with black circles were data in DBA/2J (D2) mice.

Fourteen representative gallstones or atherosclerosis relevant genes which functioned in lipid metabolism, bile acid synthesis, immune/inflammation and apoptosis were selected to validate the results of the microarray analysis. The quantitative real time PCR results exhibited a high coincidence with microarray results (Figure 3A, B, and 3C).

### TUNEL assay and Western blot

**Figure 4:**
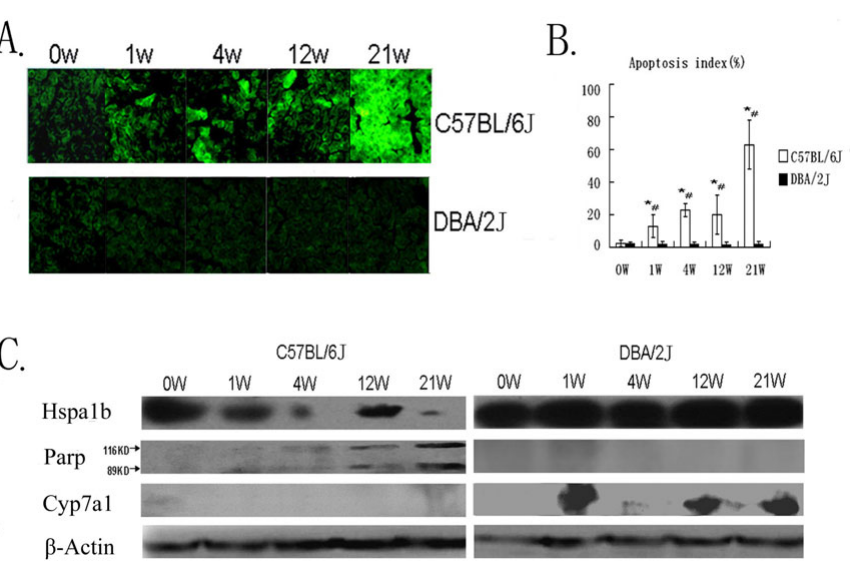
**TUNEL Assay (immunoflurescent labelling) and Western-blot analysis**. ***A*: **C57BL/6J mice, apoptotic liver cells radiating strong green light, normal cells radiating weak green light:DBA/2J mice, little apoptotic liver cells appeared. Time-course is shown from left to right; ***B*: **Apoptosis index (percentage) in C57BL/6J and DBA/2J mice. X-axis represent time-points, y-axis represents cell apoptosis percentage. *P < 0.05 (compared to 0 week), #P < 0.05(compared to DBA/2J). ***C: ***Western blot analysis for selected proteins indicated proteins expression was also differentially changed. Proteins regulation was seen for Hspa1b, Parp and Cyp7a1.Beta-Actin served as loading control.

Under a fluorescence microscope, normal cells radiated weak green light while apoptotic cells radiated strong green light. Comprehensive counts showed that apoptotic liver cells appeared increasingly in B6 mice from the 1^st ^week to the 21^st ^week, however, this observation was not found in D2 mice (Figure 4A and 4B). We also observed that more apoptotic cells occurred at the 4^th ^week when gallstones were primarily formed, and the most apoptotic cells appeared at the 21^st ^week when atherosclerotic lesions were detected.

Several apoptosis, gallstones or atherosclerosis relevant genes were selected to perform western-blot. As showed in Figure 4C, the multifunctional protein Hspa1b was clearly down-regulated in B6 mice and slightly up-regulated in D2 mice. The expression of apoptosis marker protein Parp was elevated in B6 mice and its activated form (89KD) was also observed at 1,4,12 and 21 weeks, which was unable to be detected in D2 mice. Cyp7a1, a key rate-limiting enzyme of bile acid synthesis, showed decreased expression in B6 mice but increased expression in D2 mice.

## Discussion

In the present study, we applied an animal model to delineate the different responses to a high-fat/cholesterol diet between B6 and D2 inbred mice, and uncover underlying genetic factors for these different responses.

We found an interesting and distinctive dynamic distribution of cholesterol and bile acid yields between B6 and D2 strains. We hypothesized that under the challenge of a high-fat/cholesterol diet, D2 mice, which are resistant to both gallstones and atherosclerosis, might prevent this damage through an increased bile acid synthesis approach; whereas, B6 mice might only avoid the damage by their limited direct secretion of cholesterol into bile. The hepatic genes expression profiles confirmed our hypothesis, and provided more information by identifying differentially expressed genes in pathways of lipid metabolism, oxidative stress, immune response/inflammation and apoptosis.

*Abca1*, *Abcg5 *and *Abcg8 *belong to the family of ABC transporters, which can mediate secretion of excessive cholesterol into bile and play important roles in consequent gallstones or atherosclerosis [16]. Cytochrome P450 subfamily members Cyp7a1 and Cyp8b1 were pivotal enzymes of converting cholesterol into bile acids in bile acid synthesis pathway [17]. After fed the high-fat/cholesterol diet, *Abca1*, *Abcg5 *and *Abcg8 *genes expression increased, but *Cyp7a1 *and *Cyp8b1 *decreased since the 1^st ^week in B6 mice. This indicated bile acid synthesis might be depressed, excessive cholesterol might directly excrete into bile and result in elevated bile TC in B6 mice. We observed the highest fold-changes of *Abcg5 *and *Abcg8 *at the 4^th ^week in B6, which were 9.5 and 6.3 folds respectively. The distinct up-regulation of genes *Abcg5 *and *Abcg8 *might implicate hypersecretion of cholesterol into bile and lead to gallstones formation. Interestingly, we noticed the peak of bile TC level and primary gallstones formation at the 4^th ^week. Thereafter, gallstones were increasingly formed; serum TC slightly lowered after the 12^th ^week and atherosclerotic lesions were not formed until the 21^st^week. This might indicate a potential role of gallstones formation in lowering serum cholesterol and protective effects in atherosclerosis formation. To date, the xenobiotic substrates of *Abcg5 *and *Abcg8 *have been used for regulating gallstones formation and treatments of cardiovascular disease, whereas the limited protective mechanism may not confer the expected reduction in cardiovascular risks in hypercholesterolemic patients [18]. In contrast, we observed no alteration of *Abca1*, *Abcg5 *and *Abcg8 *genes expression but increased levels of *Cyp7a1 *and *Cyp8b1 *in D2 mice. Our results suggested that excessive cholesterol might be eliminated by elevated bile acid synthesis pathway in D2 mice. To our knowledge, transgenic expression of *Cyp7a1 *might efficiently eliminate excessive cholesterol and inhibit formation of gallstones and atherosclerosis in mice [19]. In the present study, neither gallstones nor atherosclerosis was found in D2 mice. Therefore, modulation of enzymes and nuclear receptors involved in bile acid synthesis might be potential pharmaceutical targets to control hypercholesterolemia and prevent gallstones and atherosclerosis.

Modulation of *Cyp7a1 *may differ among inbred mice as a result of diverse dietary and genetic factors. It is reported that two high-fat diets contained safflower oil and 0.5% sodium cholate with and without added cholesterol (0.5%) resulted in differential expression levels of *Cyp7a1*among nine inbred mice strains [20]. It is worthwhile to note that FXR (farnesoid × receptor) and LXR (Liver × receptor) α are negative and positive regulators of *Cyp7a1*. Cholic acid component is needed for FXR activation and that cholic acid, cholesterol and high fat are necessary for activating LXRα. The different balance between LXRα and FXR actions may lead to the diverse effects of dietary cholesterol on *Cyp7a1 *expression [21]. In the present study, both the transcript and protein levels of *Cyp7a1 *decreased in B6 and increased in D2 mice, which coincided with previous study [20]. Intriguingly, we observed that FXR target gene Shp or Nr0b2 (short heterodimer partner, nuclear receptor subfamily 0, group B, member 2) was clearly increased in B6 but not altered in D2. This suggested that LXRα and FXR dominant actions might differ between B6 and D2. Thus, *Cyp7a1 *might be modulated by different nuclear receptors, resulting in diverse regulation of cholesterol and subsequently varying susceptibilities to gallstones and atherosclerosis. Besides *Cyp7a1 *and *Cyp8b1*, many other cytochrome P450 subfamily members such as *Cyp2c44*, *Cyp2c55 *and *Cyp3a13 *were also differently modulated. Further investigation in their functions and regulators might help identifying novel target genes of gallstones and atherosclerosis. In addition, genes *Soat1 *and *Soat2 *are known to encode ACAT1 and ACAT2 enzymes in catalyzing the formation of cholesteryl esters, an important process during atherosclerosis formation [22]. ACAT inhibitors have been reported to increase bile acid synthesis by up-regulation of *Cyp7a1 *expression in cultured rat hepatocytes [23]. In the present study, we observed that genes *Soat1 *and *Soat2 *were differently regulated between B6 and D2. This might provide a good demonstration that cholesteryl esters levels differed, and susceptibilities to gallstones and atherosclerosis varied between B6 and D2. Taken together, our findings suggested that the different distribution of cholesterol, and varying susceptibilities to gallstones and atherosclerosis might partly be attributable to diverse modulation of lipid metabolism or bile acid synthesis pathways.

Metabolic pathways involving cytochrome P450 enzymes may initiate or modulate oxidative damage due to oxygen radicals [24]. Moreover, oxidative stress may lead to enhanced lipid peroxidation, the generation of hydroperoxides and other toxic compounds, which may contribute to the development of gallstones and atherosclerosis [25,26]. The susceptibility to oxidative stress is reported to differ among inbred mice, which may have long-term implications in therapeutics and patient care if similar inherited differences exist in humans [24]. In the present study, abundant metabolic pathways genes as well as oxidative stress related genes were identified differently regulated between B6 and D2 mice. For instance, both mRNA and protein levels of Hspa1b were depressed in B6 and enhanced in D2 mice. Heat shock proteins have been considered to exert protective effects, due to their multifunction in anti-oxidative activity, cell survival, suppressing apoptosis, regulating pro-inflammatory transcription factors [27]. It is coincident that gallstones and atherosclerosis have been regarded as inflammatory diseases [15,28]. In parallel, multiple inflammation relevant genes were shown to represent different expression patterns between B6 and D2 mice. Icam1 (intercellular adhesion molecule1) and Vcam1 (vascular cell adhesion molecule 1) which are immunoglobin subfamily members and important inflammatory factors during the development and progression of atherosclerosis disease [29], were elevated in B6 and lowered in D2 mice. Interestingly, inflammatory responses have been reported to repress bile acid synthesis by negative regulating Cyp7a1 [30]. Altogether, after fed the high-fat/cholesterol diet, B6 and D2 mice might differ in abilities to dispose oxidative stress, induce diverse inflammatory responses and subsequent varying susceptibilities to gallstones or atherosclerosis. Our microarray data might have provided a potential genetic basis for this hypothesis, which might have long-term significance in identifying novel pharmaceutical targets in related diseases.

Cell apoptosis playing important roles in gallstones and atherosclerosis has been well delineated. Hydrophilic ursodeoxycholic acid (UDCA) exerts antiapoptotic effects and has been used as a novel therapeutic agent for the treatment of apoptosis-related liver diseases including gallstones [31]. Endothelial cell apoptosis has been reported to be responsible for the formation of coronary thrombotic atherosclerotic plaques in rabbits [32]. In the present study, microarray analysis revealed different regulation of several pro-apoptotic or apoptotic genes between B6 and D2. Furthermore, it was confirmed by QRT-PCR and western-blot analysis. Using TUNEL Assay, diverse hepatic apoptosis were observed between B6 and D2. Meanwhile, increasing apoptotic cells were concomitant with the progress of gallstones and atherosclerosis. It seemed that ambiguous relationships might exist between hepatic apoptosis and gallstones or atherosclerosis. More extensive investigation in this issue may open a new avenue to understand the mechanisms of gallstones and atherosclerosis.

## Conclusion

In all, we observed significantly different responses to a high-fat/cholesterol diet between B6 and D2 mice, including an interesting dynamic distribution of cholesterol in serum and bile, hepatic apoptosis, gallstones and atherosclerosis formation. Using genome-wide microarray analysis, hepatic genes expression profiles revealed different patterns in functional pathway groups including lipid metabolism, oxidative stress, immune/inflammation response and apoptosis, which might provide a genetic basis for diverse responses between B6 and D2 mice. This might provide us new insights into gallstones and atherosclerosis formation, and give us an opportunity to identify candidate genes in associated diseases.

## Abbreviations

B6: C57BL/6; D2: DBA/2; TUNEL: Terminal Transferase dUTP Nick End Labeling; TC: total cholesterol; HDL: high density lipoprotein; QRT-PCR: Quantitative real-time polymerase chain reaction.

## Competing interests

The authors declare that they have no competing interests.

## Authors' contributions

MZZ participated in design of this study and performed animal experiments, biochemical analysis, microarray analysis and drafted the manuscript. GZJ designed this study and interpreted data. GJ and ZBY participated in design of this study, interpretation of results and manuscript revising. All authors read and approved the final manuscript.

## Supplementary Material

Additional file 1 **Table S1, Primers for quantitative real-time-PCR**. Primers for quantitative real-time-PCR. Click here for file

Additional file 2 **Figure S1, Atherosclerotic lesions and the weight of gallstones**. Atherosclerotic lesions at the 21^st ^week and the weight of gallstones at each time-point in B6 mice. Click here for file

Additional file 3 **Table S2, Differentially expressed genes**. Log_2_ratio was calculated between 0 week and the rest of time points in each mouse strains. Click here for file

Additional file 4 **Figure S2. Function categories of further analysis genes**. Categories of functions in differentially expressed genes. Click here for file

Additional file 5 **Table S3, Detailed pathways information**. Detailed pathways identified in differentially expressed genes and detailed information of their corresponding genes. Click here for file

Additional file 6 **Figure S3. Hierarchical cluster analysis**. Hierarchical cluster analysis was performed using log_2_ratios calculating between B6 and D2 at each time point. Samples (five time points) are displayed in columns and genes are displayed in rows. Click here for file
