# Supplementary figures and images for: Different responsiveness to a high-fat/cholesterol diet in two inbred mice and underlying genetic factors: a whole genome microarray analysis
Source: Nutr Metab (Lond). 2009 Oct 17;6:43. doi: 10.1186/1743-7075-6-43 (PMC2768731; doi:10.1186/1743-7075-6-43)

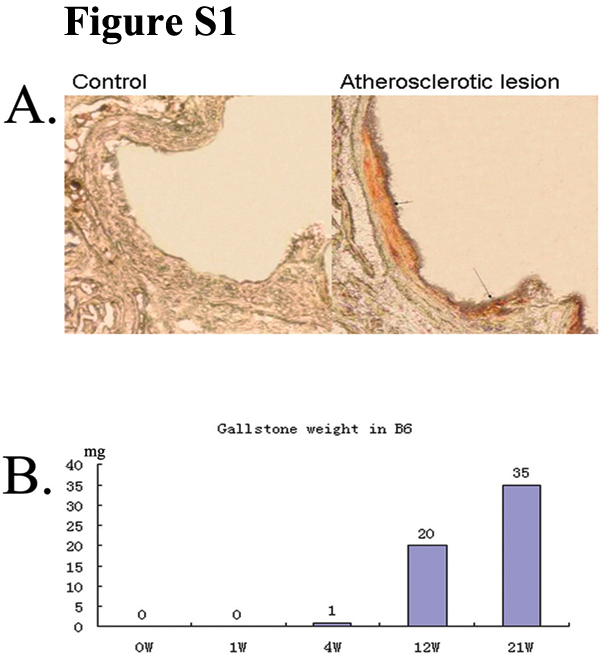

Supplement: Additional file 2 — Figure S1, Atherosclerotic lesions and the weight of gallstones. Atherosclerotic lesions at the 21st week and the weight of gallstones at each time-point in B6 mice. [file 1743-7075-6-43-S2.JPEG]

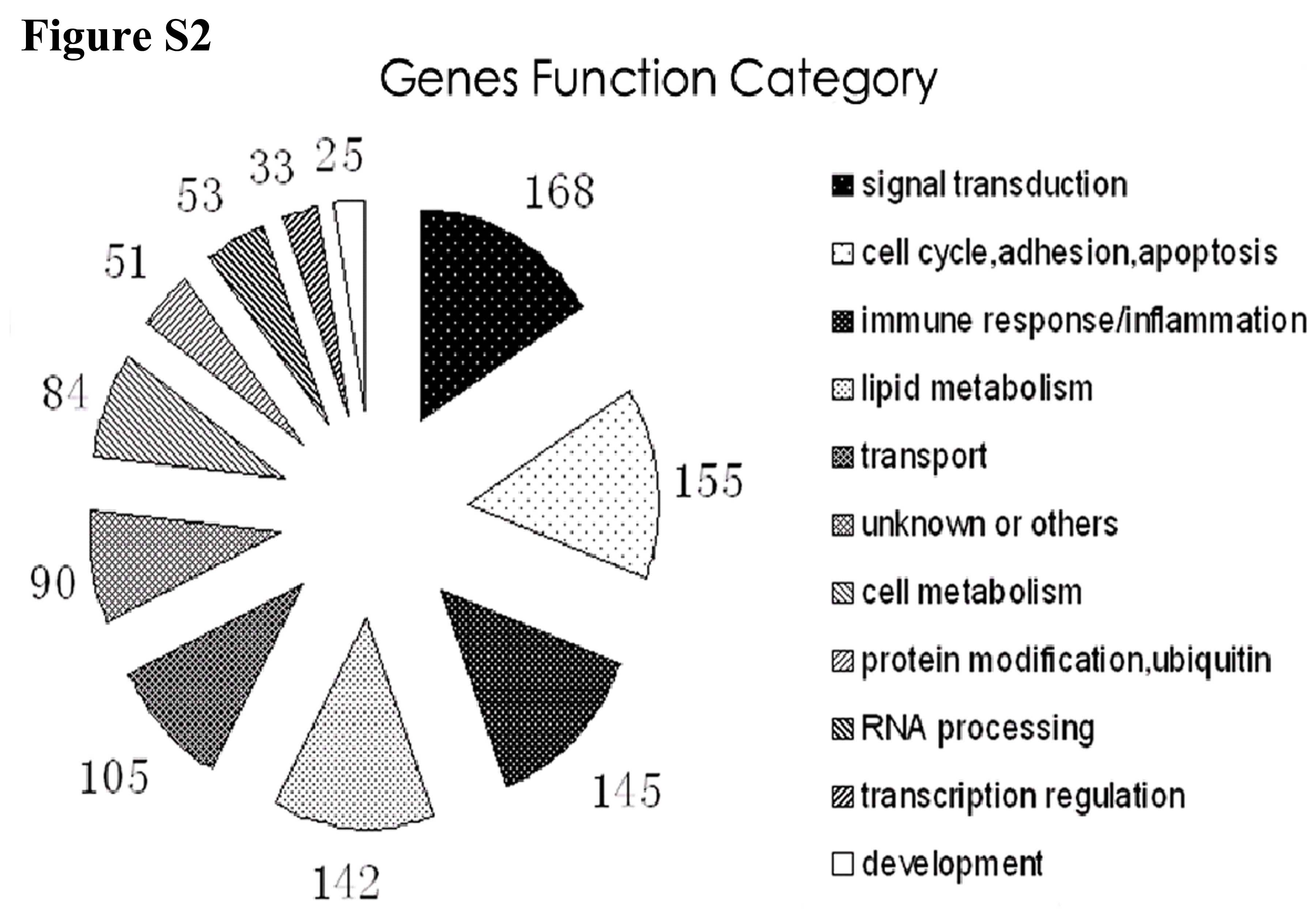

Supplement: Additional file 4 — Figure S2. Function categories of further analysis genes. Categories of functions in differentially expressed genes. [file 1743-7075-6-43-S4.JPEG]

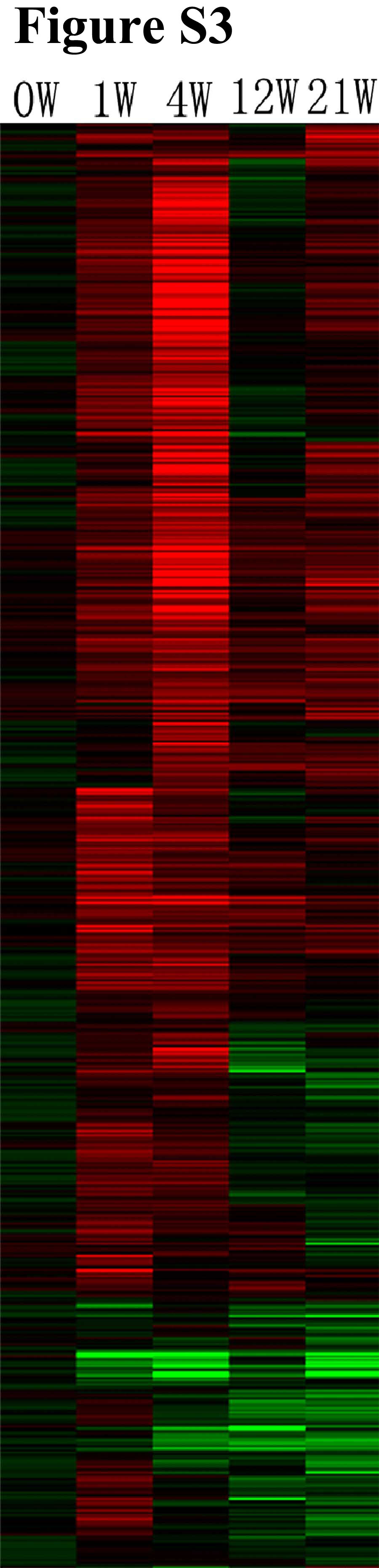

Supplement: Additional file 6 — Figure S3. Hierarchical cluster analysis. Hierarchical cluster analysis was performed using log2ratios calculating between B6 and D2 at each time point. Samples (five time points) are displayed in columns and genes are displayed in rows. [file 1743-7075-6-43-S6.JPEG]
